# AIDS with obesity, hypothyroidism and elevated serum creatinine: A case report

**DOI:** 10.3389/fmed.2023.1090659

**Published:** 2023-03-14

**Authors:** Chenxi Zhang, Chuyue Qian, Wanning Wang, Zhi Chen, Yangyang Lin, Mindan Sun

**Affiliations:** Department of Nephrology, The First Hospital of Jilin University, Changchun, China

**Keywords:** hypothyroidism, elevated serum creatinine, obesity, levothyroxine, HIV, HAART

## Abstract

Hypothyroidism is a prevalent endocrine illness with a variety of clinical symptoms, but among which elevated serum creatinine is uncommon. Hypothyroidism is also common in acquired immunodeficiency syndrome (AIDS) patients, especially those receiving highly active antiretroviral treatment (HAART). Here we present a case of a young AIDS patient with hypothyroidism, increased serum creatinine, and obesity. Despite the lack of a kidney biopsy, following levothyroxine (LT4) therapy, his serum creatinine recovered to normal levels, and weight loss, edema, weakness, rough skin and other clinical symptoms obtained notable improvement. This highlights the need of clinicians paying attention to whether thyroid function is aberrant in human immunodeficiency virus (HIV) patients with increased creatinine, edema and significant weight gain since prompt thyroid hormone therapy can restore the alterations in renal function and avoid invasive renal biopsy.

## Introduction

Hypothyroidism is a prevalent systemic metabolic condition marked by inadequate thyroid hormones secretion, and common symptoms include weakness, weight gain, chills, constipation, edema and others, but renal involvement is rare (1), which affects the kidney by decreasing renin release, increasing vascular resistance, lowering renal plasma flow, and causing renal tubular dysfunction (2). Moreover, hypothyroidism is also common in HIV patients, especially those with HAART (3). Here we present a case of an AIDS patient with hypothyroidism, elevated serum creatinine and obesity who gradually recovered to energy, physical strength and normal renal function after receiving LT4 treatment.

## Case report

A 30-year-old Chinese male was admitted to the hospital in June 2021 for weakness with edema of both lower limbs for 1 year and elevated serum creatinine for 1 week. 1 year before to admission, the patient began to experience intermittent edema in both lower limbs for no apparent reason. Because there were no noticeable abnormalities in the urine routine, renal function, or liver function throughout this time, no further examinations were conducted. 1 week ago, the patient noticed that his edema in both lower limbs had worsened, and his renal function revealed an increase in serum creatinine. Prior medical history comprised diagnosed with hypothyroidism 10 months earlier but untreated, AIDS 8 months earlier and had been receiving HAART (Tenofovir disoproxil fumarate 300 mg, Lamivudine 300 mg and efavirenz 600 mg). The most recent CD4^+^ T lymphocyte count was 414.1 /μL (normal range: 346.4–985.1 /μL), while HIV-RNA was negative.

The main clinical manifestations included weakness, fatigue, hypomnesis, slow movement, chills, thirst, poor appetite, drowsiness with greater snoring, and constipation. Physical examination showed dull complexion, lags in response, slow speech, rough skin, and edema in both lower limbs. His heart rate and blood pressure and body mass index (BMI) were 75 bpm, 135/86 mm/Hg and 33.3 kg/m^2^ (body weight: 102 kg; body length: 1.75 m), respectively. Laboratory tests revealed that serum creatinine 1.43 mg/dL (normal: 0.64–1.10), proteinuria 0.00 g/24 h (normal: <0.15), creatine kinase (CK) 289 U/L (normal: 25–200 U/L), aspartate aminotransferase (AST) 83.1 U/L (normal range 15.0–40.0) and alanine aminotransferase (ALT) 64.8 U/L (normal range 9.0–50.0), serum albumin 47.0 g/L (normal: 40.0–55.0), and hemoglobin 108 g/L (normal: 130–175). The thyroid function test marked severe hypothyroidism: thyroid-stimulating hormone (TSH) 71.36 μIU/mL (normal values: 0.35–4.94), free triiodothyronine (FT3) < 1.64 pmol/L (normal: 2.43–6.01), free thyroxine (FT4) <5.15 pmol/L (normal: 9.01–19.05), anti-thyroglobulin antibodies (TG-Ab) >1000.00 IU/mL (normal: 0–4.11), anti-thyroid peroxidase antibodies (TPO-Ab) >1000.00 IU/mL (normal: 0–5.61). Additionally, routine urine examination, serological tests for antinuclear antibodies, anti-double-strand DNA antibodies, anti-neutrophil cytoplasmic antibodies and anti-phospholipase A2 receptor antibodies were all negative. Meanwhile, imageological examination detected left atrial and left ventricular enlargement, pericardial effusion, bilateral pleural effusion and diffuse ultrasonographic changes in thyroid parenchyma with a high possibility of hypothyroidism. The results of the abdominal color doppler ultrasonography were normal, and the size of the left kidney was 100*54 mm as well as the right kidney was 105*48 mm. We recommended a renal biopsy to determine the cause of the patient’s illness, but he ultimately declined due to the associated risks and concerning about how much impact kidney biopsy made in therapy.

**Figure 1:**
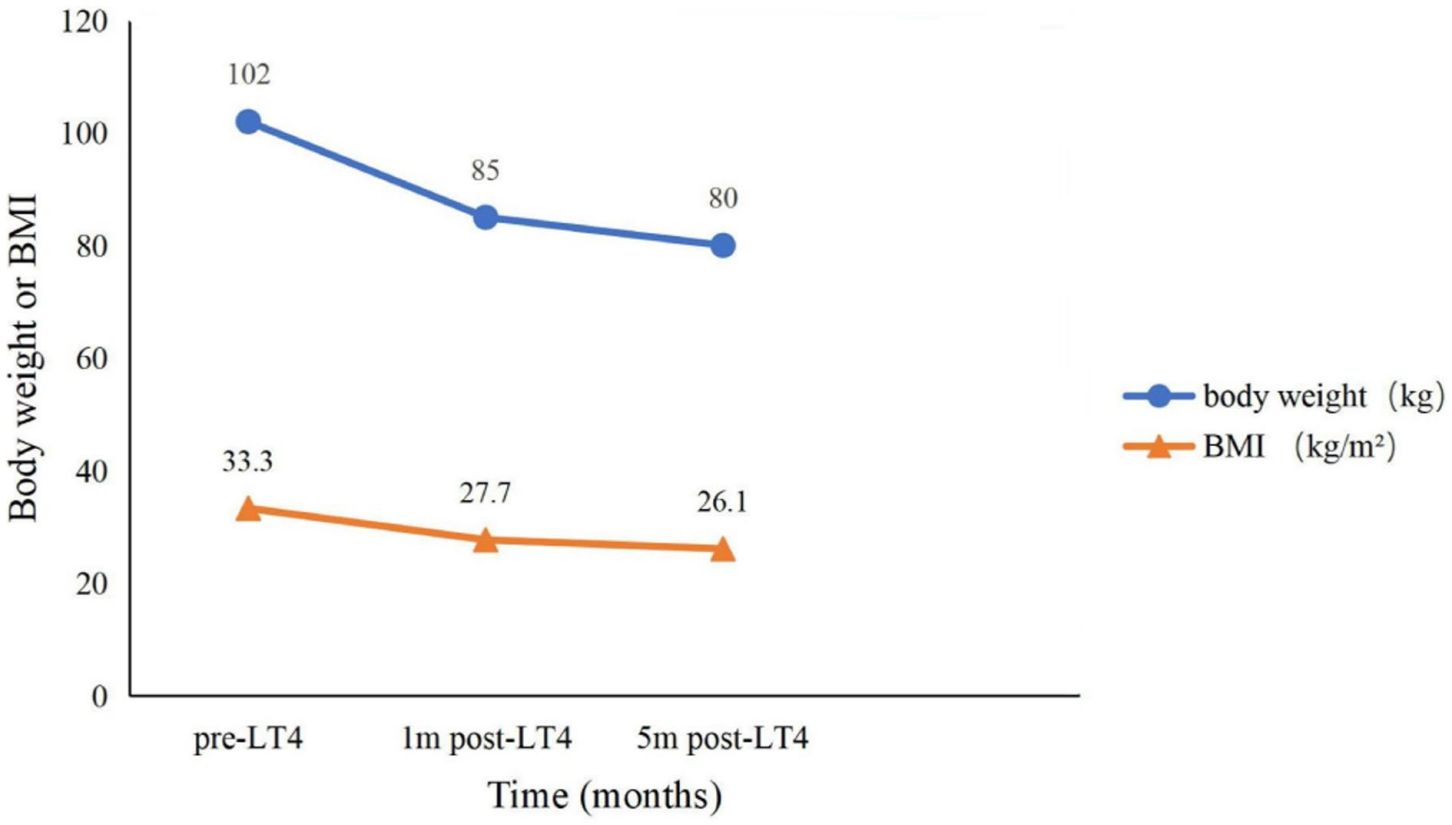
Trends in body weight and BMI over time. Pre-LT4, before levothyroxine treatment; 1 m post-LT4, 1 month after levothyroxine treatment; 5 m post-LT4, 5 months after levothyroxine treatment; BMI, body mass index.

Combined with the patient’s main symptoms and thyroid function tests, the diagnosis of hypothyroidism was confirmed. After admission, we administered LT4 50 μg orally once daily for 3 days, and then adjusted the dose to 75 μg. During the follow-up, the main abnormal laboratory parameters gradually improved (Table 1). 1 month later, the patient’s body weight decreased to 85 kg (BMI 27.7 kg/m^2^), edema was lightened, serum creatinine returned to normal (1.09 mg/dL). Then 2 months later, besides his response lags, slow speech, chills, weakness, fatigue, and roughness of the skin had significantly improved, edema disappeared and liver function normalized, he took on a completely new look compared to his admission. After 5 months of treatment, his weight dropped to 80 kg (BMI 26.1 kg/m^2^); (Figure 1), hemoglobin returned to normal, and the LT4 dose increased from 75 μg to 125 μg. The follow-up conducted until now, his serum creatinine levels had been in the normal range and thyroid function tests were nearly normal. Meanwhile he was also in stable condition with AIDS with no opportunistic infections.

**Table 1:** Main laboratory tests during admission and follow-up.

Time since levothyroxine					
laboratory tests	0 month	1 month	2 months	5 months	7 months
creatinine (mg/dL)	1.43	1.09	0.93	0.73	0.73
proteinuria (g/24 h)	0.00	–	0.05	0.10	–
HGB (g/L)	108	110	120	135	141
AST (U/L)	83.1	41.7	25.5	23.6	27.7
ALT (U/L)	64.8	44.7	34.4	26.0	32.3
TSH (μIU/mL)	71.36	49.53	–	19.09	–
FT3 (pmol/L)	< 1.64	2.15	–	3.49	–
FT4 (pmol/L)	< 5.15	5.18	–	9.71	–
TPO-Ab (IU/mL)	>1000.00	–	–	–	–
TG-Ab (IU/mL)	>1000.00	–	–	–	–

Creatinine (0.64–1.10 mg/dL); proteinuria (<0.15 g/24 h); HGB, hemoglobin (130–175 g/L); AST, aspartate aminotransferase (15.0–40.0 U/L); ALT, alanine aminotransferase (9.0–50.0 U/L); TSH, thyroid-stimulating hormone (0.35–4.94μIU/mL); FT3, free triiodothyronine (2.43–6.01 pmol/L); FT4, free thyroxine (9.01–19.05 pmol/L); TPO-Ab, anti-thyroid peroxidase antibodies (0–4.11 IU/mL); TG-Ab, anti-thyroglobulin antibodies (0–5.61 IU/mL).

## Discussion

For one thing, thyroid hormones influence kidney development and function in both direct and indirect ways, including influencing the size and structure of the fetal kidney, directly affecting renal blood flow (RBF), glomerular filtration rate (GFR), and renal tubular secretion and reabsorption function, and indirectly influencing kidney function by regulating systemic vascular resistance and cardiac output (4). Previous studies have shown that hypothyroidism can cause elevated CK and serum creatinine, proteinuria, even acute kidney injury (AKI), and the underlying mechanisms of associated renal injury include reduced myocardial contractility, increased peripheral vascular resistance, decreased cardiac output, decreased renal vasoconstriction and vasodilator synthesis, and structural pathological changes such as glomerular basement membrane thickening and mesangial matrix increasing, leading to decreased RBF and GFR (5–9). Pathological categories of hypothyroid-related renal damage shown by renal biopsy included membranous glomerulonephritis, focal segmental glomerulosclerosis, minimal change disease, membranoproliferative glomerulonephritis and so on (10).

In rare cases, severe hypothyroidism can induce rhabdomyolysis leading to AKI (7, 11, 12). But rhabdomyolysis is ruled out in our instance due to the absence of muscular discomfort and tenderness, a normal urine test, and, most critically, a CK value less than 5 times the upper limit of the normal range. In addition, the main renal manifestations of this patient were elevated serum creatinine without hematuria or proteinuria, color Doppler ultrasound revealed that both kidneys were normal in size, and serum creatinine declined to the normal range after 1 month, which was not consistent with typical nephritis or chronic kidney disease (CKD). It was surprising that this patient experienced reversible alterations in renal function following LT4 treatment. And Gou et al. also obtained comparable results to those indicated above (6, 7, 13). In a prospective severe hypothyroidism research, thyroid hormone replacement treatment could lower urine protein excretion while improving estimated GFR (14).

Hypothyroidism is also a risk factor for the occurrence and progression of CKD (8), increasing the hazard of mortality in the dialysis population (15). Although it is debatable whether subclinical hypothyroidism (SCH) necessitates thyroid hormone therapy, previous research has indicated that LT4 therapy helps protect renal function in CKD with SCH (16).

For another thing, HIV is a critical component that must not be overlooked. Hypothyroidism is common in the AIDS community, nevertheless, the pathophysiology is still unknown at present, which seems to be related to immune activation triggered by opportunistic infections (17). Ji et al. discovered that FT3 and FT4 levels were adversely connected to HIV duration and favorably related to CD4 cell counts (18). In addition, Patients on HAART had a higher prevalence of hypothyroidism and a lower level of FT4, with the likely cause being that HAART that HAART induced autoimmunity in the process of immunological reconstitution by suppressing HIV replication and increasing the number of CD4 positive memory and naive cells (3, 18). And TSH levels increased with the duration of HAART (19). This patient was in the asymptomatic stage of HIV infection with no other opportunistic infections. He had been on HAART and evaluated on a regular basis prior to admission. Even though the diagnosis of hypothyroidism came before the diagnosis of HIV and the initiation of HAART, the role of HIV and HAART in the development of hypothyroidism cannot be ruled out. As a result, clinicians should be on the high alert for thyroid dysfunction in AIDS patients with unexplained increased serum creatinine. We urge that HIV patients, particularly those on HAART, have their thyroid function checked on a frequent basis.

Furthermore, the patient in question is an obese guy with a BMI of 33.3 kg/m^2^. TSH levels that are consistently high may cause chronic low-grade inflammation, increasing leptin and inflammatory factor release, resulting in adipose tissue malfunction and weight gain (20). Obesity can affect thyroid function as well, the mechanisms of which are uncertain and may be linked to inflammatory factors reducing the iodine uptake activity of thyroid cells by inhibiting the mRNA expression of symporter sodium/iodide, regulating the expression of deiodinase and adipokines such as leptin inhibiting thyroglobulin expression to affect thyroid function according to researches (20, 21). As a result, we also counseled the patient to lose weight throughout LT4 treatment by food and lifestyle adjustments in order to attain a better prognosis, and the patient’s weight steadily reduced during follow-up.

## Conclusion

Hypothyroidism can cause serious weakness, increase in serum creatinine, and considerable weight gain. In severe situations, it can render patients unable to carry out everyday tasks and drastically degrade their quality of life, resulting in a devastating physiological and psychological blow to patients. We suggest that clinicians should be alert to abnormal thyroid function when they encounter elevated serum creatinine. Because if hypothyroidism is corrected, blood creatinine levels can return to normal in some individuals, and weight loss, energy, and physical strength can all be greatly improved, allowing patients to not only return to society but also avoid invasive kidney biopsy to achieve a good prognosis.

## Data availability statement

The original contributions presented in the study are included in the article/supplementary material, further inquiries can be directed to the corresponding author.

## Ethics statement

Written informed consent was obtained from the individual(s) for the publication of any potentially identifiable images or data included in this article.

## Author contributions

CZ, CQ, and YL gathered clinical data. CZ and CQ analyzed the data and wrote the manuscript. MS, WW, and ZC revised the content. All authors contributed to the article and approved the submitted version.

## Conflict of interest

The authors declare that the research was conducted in the absence of any commercial or financial relationships that could be construed as a potential conflict of interest.

## Publisher’s note

All claims expressed in this article are solely those of the authors and do not necessarily represent those of their affiliated organizations, or those of the publisher, the editors and the reviewers. Any product that may be evaluated in this article, or claim that may be made by its manufacturer, is not guaranteed or endorsed by the publisher.
